# Supplementary figures and images for: Genetic Determinants of Atherogenic Indexes
Source: Genes (Basel). 2023 Jun 1;14(6):1214. doi: 10.3390/genes14061214 (PMC10298447; doi:10.3390/genes14061214)

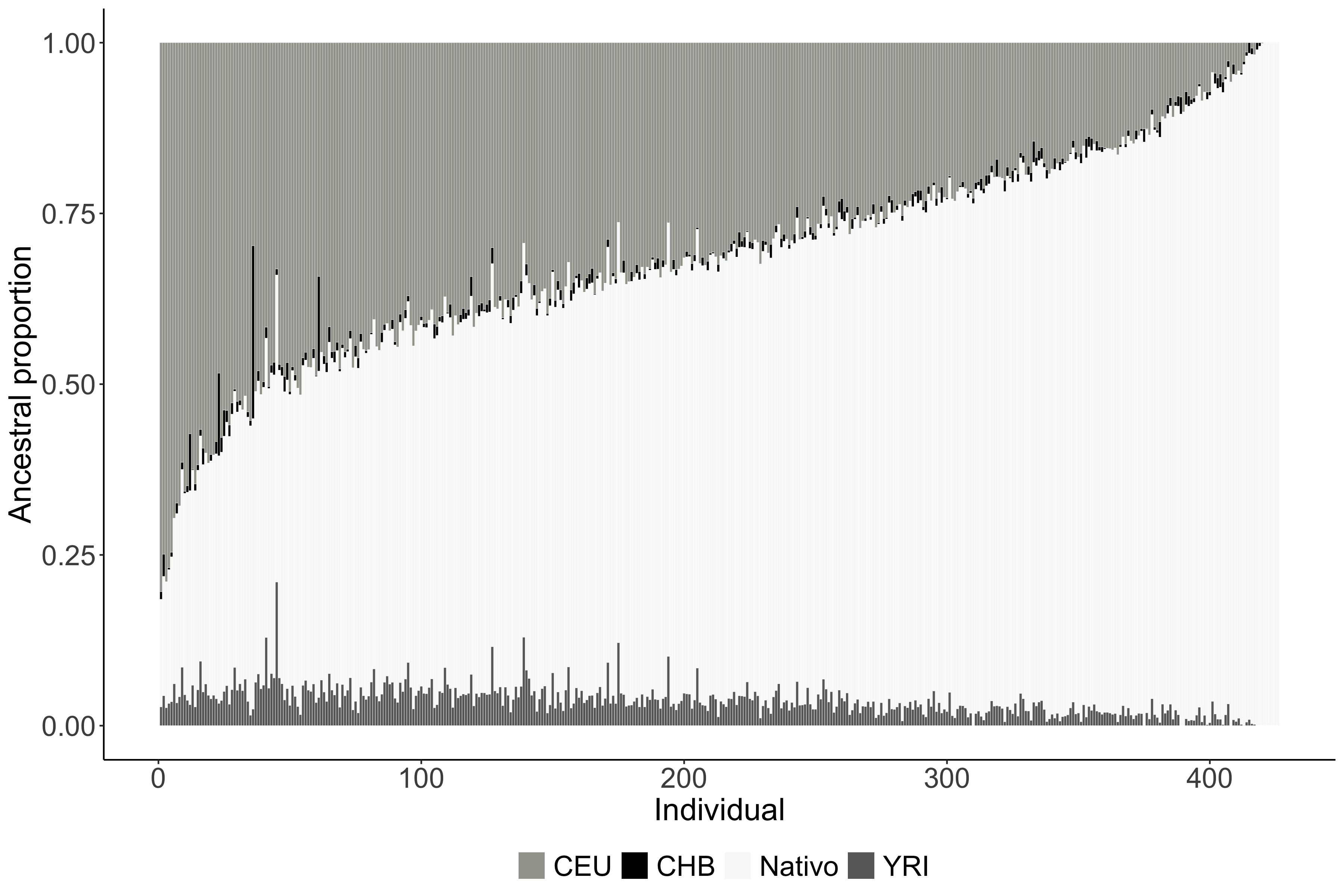

Supplement: Supplementary file 1 [file genes-14-01214-s001.zip › genes-2372416-supplementary/FigureS1.png]
